# Supplementary material for: Variational consistent histories as a hybrid algorithm for quantum foundations
Source: Nat Commun. 2019 Jul 31;10:3438. doi: 10.1038/s41467-019-11417-0 (PMC6668436; doi:10.1038/s41467-019-11417-0)
Supplement: Supplementary file 1 — Supplementary Information [file 41467_2019_11417_MOESM1_ESM.pdf]

Supplementary Information–
Variational Consistent Histories as a Hybrid Algorithm for Quantum Foundations

Arrasmith et al.

SUPPLEMENTARY NOTE 1 – GENERALIZATIONS

Here we discuss various generalizations of the circuits shown in the main text, which presented our VCH algorithm for the special case of branch-independent histories of a one-qubit system S with no environment E.

Multi-Qubit Systems

The circuits in the main text showed systems S composed of a single qubit. The generalization to multi-qubit systems is straightforward. We must discuss the generalizations of both the state preparation circuit in Fig. 2 as well as the cost evaluation circuits in Fig. 5.

Supplementary Figure 1 illustrates how the state preparation circuit generalizes to multi-qubit systems. In particular, this figure shows how a portion of state preparation circuit (the portion that entangles the system to the ancillas) generalizes for the case of a fine-grained set of projectors. (Note that the case of a coarse-grained set of projectors is discussed in the next subsection.)

The cost evaluation circuits in Fig. 5 generalize as follows. For fine-grained histories, one needs n ancillas for each time step and hence a total of nk ancillas. The circuits in Fig. 5 shown for k ancillas generalize in a straightforward way, where now one has nk ancilla systems. In addition, the circuits in Fig. 5**b** also involve the S system, and hence all n qubits in S must be included in this circuit. Again, these n qubits are included in the most straightforward way (in the same way that the single qubit S system appears in the circuits in Fig. 5**b**).

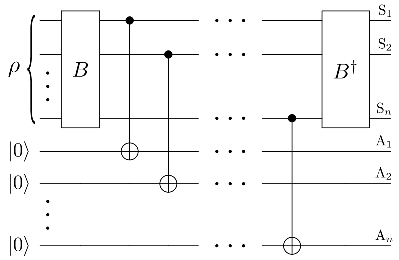

Supplementary Figure 1. The generalization of our state preparation circuit to multi-qubit systems S. In this example, we show the portion of the circuit that entangles the system and the ancillas, for the special case of a fine-grained set of projectors. In this fine-grained case, one employs the same number of ancilla qubits as are in S, i.e., n qubits.

Coarse Grained Histories

Multi-qubit systems S allow for non-trivial coarse-grained histories. In such families of histories, the sets P_j are composed of projectors whose ranks are possibly greater than one. We remark that coarse-grained histories are often important to the study of macroscropic systems and the quantum-to-classical transition. VCH can easily be adapted to study coarse-grained histories as follows.

For each time t_j, one should decide (prior to running VCH) projector ranks that one is interested in. VCH will then optimize over sets of projectors with these particular ranks. The projector ranks can therefore be viewed as hyperparameters, i.e., parameters that one fixes for a given run of VCH.

For instance, suppose S is composed of a pair of spins. In this case, Supplementary Figure 2 shows two examples of the state preparation circuit for a single time step. In the first example, Supplementary Figure 2**a**, we consider a projector set that contains two rank-two projectors revealing whether the spins were aligned or anti-aligned. In the second example, Supplementary Figure 2**b**, we consider a projector set that contains a rank-three and a rank-one projector that respectively indicate whether the spins are in the triplet states or the the singlet state. Note that the ranks of the projectors are determined by the gate that entangles the system to the ancilla, which is a single CNOT gate in Supplementary Figure 2**a** and a Toffoli gate in Supplementary Figure 2**b**. Hence the choice of the projector ranks (mentioned in the previous paragraph) translates into a choice of gate sequence that entangles the system to the ancilla.

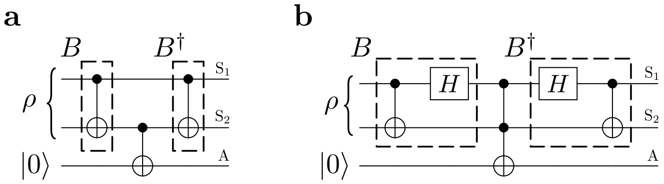

Supplementary Figure 2. Examples of implementing coarse-grained projector sets in our state preparation circuit, when S corresponds to two spin-1/2 particles. The projectors in **a** record whether the two spins are aligned or anti-aligned, while the projectors in **b** differentiate between the spin singlet and spin triplet states.

Nontrivial Environments

For many applications of VCH, (e.g., the chiral molecule example in the main text) it will be helpful to explicitly model an environment E. We can think of this case as a particular choice of coarse graining where the projectors we consider only act on a subsystem of our model (the S system) and do not directly record any information about E. Note that the Hamiltonian evolution involves both S and E, as shown in Supplementary Figure 3.

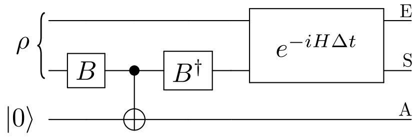

Supplementary Figure 3. Simple example with an environment E. The projectors still only act on S, but the evolution includes both S and E.

Branch Dependent Histories

A final generalization that we consider are families of branch dependent histories [1], or histories where the projector set at a given time may depend on the properties of the system at earlier points in the histories. VCH can accommodate these histories, as follows.

The basic idea is that the unitary gate B_j that determines the projector set at time t_j now becomes a controlled unitary. Specifically, the control system(s) for B_j are (potentially) all the ancilla qubits associated with times $t_i < t_j$. So the choice of projector set at some time is influenced by the ancilla states for earlier times.

Supplementary Figure 4 shows an example of what this looks like, for the special case of only two times. In this figure, if the first ancilla is in the $|0\rangle$ state ($|1\rangle$ state), then the B_2 unitary ($B_2' B_2$ unitary) is applied at the second time step. For more general cases, the B_2' unitary shown here would be replaced by a sequence of controlled unitaries controlled by different ancilla qubits.

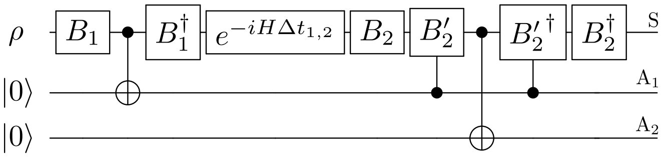

Supplementary Figure 4. Example implementation of a branch dependent projector set in our state preparation circuit. In this circuit, depending upon the result for t_1, either B_2 or the product $B_2' B_2$ defines the projector set for the second time.

SUPPLEMENTARY NOTE 2 – GENERALIZED STATE PREPARATION

We now present the details of our generalized state preparation circuit (as shown in Supplementary Figure 5) and show that σ^{SA} and σ^{A} have the properties we claim in the main text. Note that our treatment here includes all of the generalizations discussed above in SUPPLEMENTARY NOTE 1. We begin with the input state $\rho^{\mathrm{SE}} \otimes |\mathbf{0}\rangle\langle\mathbf{0}|^{\mathrm{A}}$ (where the superscript SE denotes the system and its environment and A denotes the ancillas). We then apply the gate sequence associated with the P_1 projector set, which includes B_1, a multi-qubit gate that entangles S and A (which we refer to as the "entangling gate"), and then $B_1^\dagger$. This gives the state:

$$\sum_{\alpha_1,\alpha_1'} \left[P_1^{\alpha_1} \rho^{\mathrm{SE}} P_1^{\alpha_1'\dagger}\right] \otimes \left[|\alpha_1\rangle\langle\alpha_1'| \otimes |\mathbf{0}\rangle\langle\mathbf{0}|\right]^{\mathrm{A}}. \quad (1)$$

Note that the system and ancilla are (possibly) entangled at this point.

Next in our state preparation circuit is the time evolution from t_1 to t_2, given by $e^{-iH\Delta t_{1,2}}$. This is followed by the gate sequence associated with P_2, which in general may be branch dependent. The resulting state is

$$\sum_{\alpha_1,\alpha_1',\alpha_2,\alpha_2'} \Big[P_2^{\alpha_2}(\alpha_1) e^{-iH\Delta t_{1,2}} P_1^{\alpha_1} \rho^{\mathrm{SE}} P_1^{\alpha_1'\dagger} e^{iH\Delta t_{1,2}} P_2^{\alpha_2'\dagger}(\alpha_1)\Big] \otimes \Big[|\alpha_1\rangle\langle\alpha_1'| \otimes |\alpha_2\rangle\langle\alpha_2'| \otimes |\mathbf{0}\rangle\langle\mathbf{0}|\Big]^{\mathrm{A}}, \quad (2)$$

where the notation $P_2^{\alpha_2}(\alpha_1)$ indicates that the second projector set depends on α_1. Repeating this state evolution until we have applied the gate sequences associated with all k projector sets (and switching to the Heisenberg picture), we end up with

$$\sum_{\boldsymbol{\alpha},\boldsymbol{\alpha}'} \Big[P_{\mathrm{k}}^{\alpha_{\mathrm{k}}}(t_{\mathrm{k}}) \dots P_2^{\alpha_2}(t_2) P_1^{\alpha_1}(t_1) \rho^{\mathrm{SE}} P_1^{\alpha_1'}(t_1)^\dagger P_2^{\alpha_2'}(t_2)^\dagger \dots P_{\mathrm{k}}^{\alpha_{\mathrm{k}}'}(t_{\mathrm{k}})^\dagger\Big] \otimes \Big[(|\alpha_1\rangle\langle\alpha_1'|) \otimes (|\alpha_2\rangle\langle\alpha_2'|) \otimes \dots \otimes (|\alpha_{\mathrm{k}}\rangle\langle\alpha_{\mathrm{k}}'|)\Big]^{\mathrm{A}} = \sum_{\boldsymbol{\alpha},\boldsymbol{\alpha}'} \mathcal{C}^{\boldsymbol{\alpha}} \rho^{\mathrm{SE}} \mathcal{C}^{\boldsymbol{\alpha}'\dagger} \otimes (|\boldsymbol{\alpha}\rangle\langle\boldsymbol{\alpha}'|)^{\mathrm{A}} \quad (3)$$

Note that we have suppressed explicit branch dependence here to simplify notation. Branch dependence does not alter the formalism except to make the later projectors functions of the earlier α_i's, so our treatment remains fully general.

If we then trace out the environment (which in the circuit means not measuring it) we are then left with σ^{SA}:

$$\sigma^{\mathrm{SA}} = \sum_{\boldsymbol{\alpha},\boldsymbol{\alpha}'} \mathrm{Tr}_{\mathrm{E}}(\mathcal{C}^{\boldsymbol{\alpha}} \rho^{\mathrm{SE}} \mathcal{C}^{\boldsymbol{\alpha}'\dagger}) \otimes (|\boldsymbol{\alpha}\rangle\langle\boldsymbol{\alpha}'|)^{\mathrm{A}}. \quad (4)$$

By examining Supplementary Equation (4), we can see that $(\mathbb{1} \otimes \langle\boldsymbol{\alpha}|)\sigma^{\mathrm{SA}}(\mathbb{1} \otimes |\boldsymbol{\alpha}\rangle)$ is precisely $\mathcal{D}_{\mathrm{pt}}(\boldsymbol{\alpha},\boldsymbol{\alpha}') = \mathrm{Tr}_{\mathrm{E}}(\mathcal{C}^{\boldsymbol{\alpha}} \rho^{\mathrm{SE}} \mathcal{C}^{\boldsymbol{\alpha}'\dagger})$. Further, if we similarly trace over the system S, we get:

$$\sigma^{\mathrm{A}} = \sum_{\boldsymbol{\alpha},\boldsymbol{\alpha}'} \mathrm{Tr}(\mathcal{C}^{\boldsymbol{\alpha}} \rho^{\mathrm{SE}} \mathcal{C}^{\boldsymbol{\alpha}'\dagger})(|\boldsymbol{\alpha}\rangle\langle\boldsymbol{\alpha}'|)^{\mathrm{A}}. \quad (5)$$

We can thus see that we have prepared a density matrix whose elements are $\mathcal{D}(\boldsymbol{\alpha},\boldsymbol{\alpha}') = \mathrm{Tr}(\mathcal{C}^{\boldsymbol{\alpha}} \rho^{\mathrm{SE}} \mathcal{C}^{\boldsymbol{\alpha}'\dagger})$, as claimed in the main text.

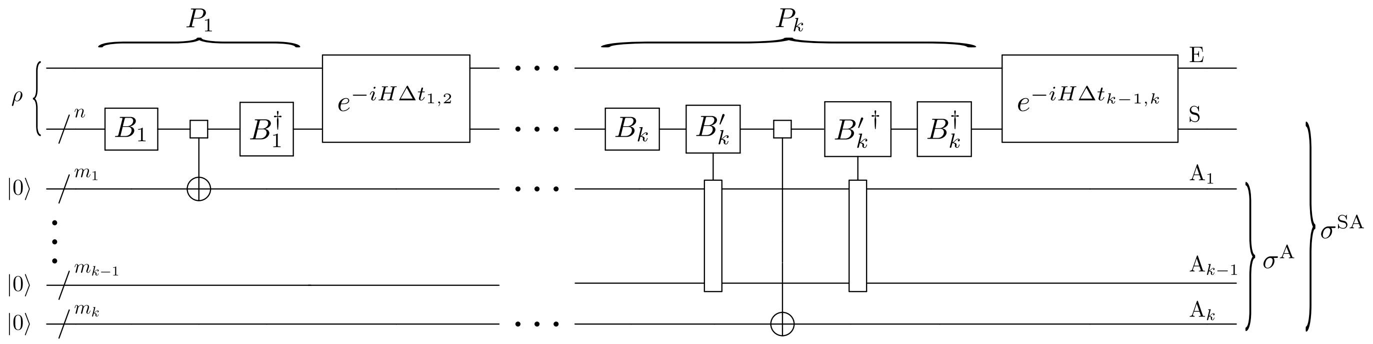

Supplementary Figure 5. The generalized state preparation circuit. A similar circuit is included as part of the flowchart, but this version incorporates larger systems and branch dependence explicitly. The multiqubit gate denotes a set of entangling gates controlled on the standard basis of the system qubits, while B' represents a parameterized unitary acting on the system controlled on the standard basis of the ancilla qubits.

SUPPLEMENTARY NOTE 3 – DERIVATION OF COST FUNCTIONS

Full trace cost

Let us now derive the equivalence stated in the definition of our full trace cost function, Eq. (9). Starting with the definition of C we have:

$$
\begin{aligned}
C &:= \sum_{\boldsymbol{\alpha}\neq\boldsymbol{\alpha}'} |\mathcal{D}(\boldsymbol{\alpha},\boldsymbol{\alpha}')|^2 \\
&= \sum_{\boldsymbol{\alpha}\neq\boldsymbol{\alpha}'} \langle\boldsymbol{\alpha}|\sigma^{\mathrm{A}}|\boldsymbol{\alpha}'\rangle\langle\boldsymbol{\alpha}'|\sigma^{\mathrm{A}}|\boldsymbol{\alpha}\rangle \\
&= \sum_{\boldsymbol{\alpha}\neq\boldsymbol{\alpha}'} \mathrm{Tr}\left((|\boldsymbol{\alpha}\rangle\langle\boldsymbol{\alpha}|)\sigma^{\mathrm{A}}(|\boldsymbol{\alpha}'\rangle\langle\boldsymbol{\alpha}'|)\sigma^{\mathrm{A}}\right) \\
&= \sum_{\boldsymbol{\alpha},\boldsymbol{\alpha}'} \mathrm{Tr}\left((|\boldsymbol{\alpha}\rangle\langle\boldsymbol{\alpha}|)\sigma^{\mathrm{A}}(|\boldsymbol{\alpha}'\rangle\langle\boldsymbol{\alpha}'|)\sigma^{\mathrm{A}}\right) \\
&\quad - \sum_{\boldsymbol{\alpha}} \mathrm{Tr}\left((|\boldsymbol{\alpha}\rangle\langle\boldsymbol{\alpha}|)\sigma^{\mathrm{A}}(|\boldsymbol{\alpha}\rangle\langle\boldsymbol{\alpha}|)\sigma^{\mathrm{A}}\right) \\
&= \mathrm{Tr}((\sigma^{\mathrm{A}})^2) - \mathrm{Tr}(\mathcal{Z}^{\mathrm{A}}(\sigma^{\mathrm{A}})^2) \\
&= \mathrm{D}_{\mathrm{HS}}(\sigma^{\mathrm{A}}, \mathcal{Z}^{\mathrm{A}}(\sigma^{\mathrm{A}})).
\end{aligned}
\tag{6}
$$

Therefore, the circuits we use to calculate $\mathrm{Tr}((\sigma^{\mathrm{A}})^2)$ and $\mathrm{Tr}(\mathcal{Z}^{\mathrm{A}}(\sigma^{\mathrm{A}})^2)$ implement this cost function as claimed.

Partial trace cost

Arriving at the expression for the partial trace cost function (Eq. (10)) is similar if slightly more complicated:

$$
\begin{aligned}
\mathrm{C}_{\mathrm{pt}} &:= \sum_{\boldsymbol{\alpha}\neq\boldsymbol{\alpha}'} \|\mathcal{D}_{\mathrm{pt}}(\boldsymbol{\alpha},\boldsymbol{\alpha}')\|_{\mathrm{HS}}^2 \\
&= \sum_{\boldsymbol{\alpha}\neq\boldsymbol{\alpha}'} \mathrm{Tr}_{\mathrm{S}}\left(\mathcal{D}_{\mathrm{pt}}(\boldsymbol{\alpha},\boldsymbol{\alpha}')\mathcal{D}_{\mathrm{pt}}(\boldsymbol{\alpha},\boldsymbol{\alpha}')^\dagger\right) \\
&= \sum_{\boldsymbol{\alpha}\neq\boldsymbol{\alpha}'} \mathrm{Tr}_{\mathrm{S}}\Big((\mathbb{1}\otimes\langle\boldsymbol{\alpha}|)(\mathbb{1}\otimes|\boldsymbol{\alpha}\rangle\langle\boldsymbol{\alpha}|)\sigma^{\mathrm{SA}} \\
&\qquad\qquad (\mathbb{1}\otimes|\boldsymbol{\alpha}'\rangle\langle\boldsymbol{\alpha}'|)\sigma^{\mathrm{SA}}(\mathbb{1}\otimes|\boldsymbol{\alpha}\rangle)\Big) \\
&= \sum_{\boldsymbol{\alpha}\neq\boldsymbol{\alpha}'} \mathrm{Tr}\left((\mathbb{1}\otimes|\boldsymbol{\alpha}\rangle\langle\boldsymbol{\alpha}|)\sigma^{\mathrm{SA}}(\mathbb{1}\otimes|\boldsymbol{\alpha}'\rangle\langle\boldsymbol{\alpha}'|)\sigma^{\mathrm{SA}}\right) \\
&= \sum_{\boldsymbol{\alpha},\boldsymbol{\alpha}'} \mathrm{Tr}\left((\mathbb{1}\otimes|\boldsymbol{\alpha}\rangle\langle\boldsymbol{\alpha}|)\sigma^{\mathrm{SA}}(\mathbb{1}\otimes|\boldsymbol{\alpha}'\rangle\langle\boldsymbol{\alpha}'|)\sigma^{\mathrm{SA}}\right) \\
&\quad - \sum_{\boldsymbol{\alpha}} \mathrm{Tr}\left((\mathbb{1}\otimes|\boldsymbol{\alpha}\rangle\langle\boldsymbol{\alpha}|)\sigma^{\mathrm{SA}}(\mathbb{1}\otimes|\boldsymbol{\alpha}\rangle\langle\boldsymbol{\alpha}|)\sigma^{\mathrm{SA}}\right) \\
&= \mathrm{Tr}((\sigma^{\mathrm{SA}})^2) - \mathrm{Tr}(\mathcal{Z}^{\mathrm{A}}(\sigma^{\mathrm{SA}})^2) \\
&= \mathrm{D}_{\mathrm{HS}}(\sigma^{\mathrm{SA}}, \mathcal{Z}^{\mathrm{A}}(\sigma^{\mathrm{SA}})).
\end{aligned}
\tag{7}
$$

As with the full trace cost function, the circuits we use to calculate $\mathrm{Tr}((\sigma^{\mathrm{SA}})^2)$ and $\mathrm{Tr}(\mathcal{Z}^{\mathrm{A}}(\sigma^{\mathrm{SA}})^2)$ thus implement this cost function as claimed.

SUPPLEMENTARY NOTE 4 – READING OUT THE DECOHERENCE FUNCTIONAL ELEMENTS

While VCH avoids the need to compute the exponentially many $\mathcal{D}(\boldsymbol{\alpha},\boldsymbol{\alpha}')$'s in order to determine the consistency of a family $\mathcal{F}$, we do have the ability to efficiently read out any particular $\mathcal{D}(\boldsymbol{\alpha},\boldsymbol{\alpha}')$ if desired. Supplementary Figure 6 shows the circuit that one can use to read the real and/or imaginary parts of $\mathcal{D}(\boldsymbol{\alpha},\boldsymbol{\alpha}')$ out for $\boldsymbol{\alpha}\neq\boldsymbol{\alpha}'$. The post-processing is similar to that of

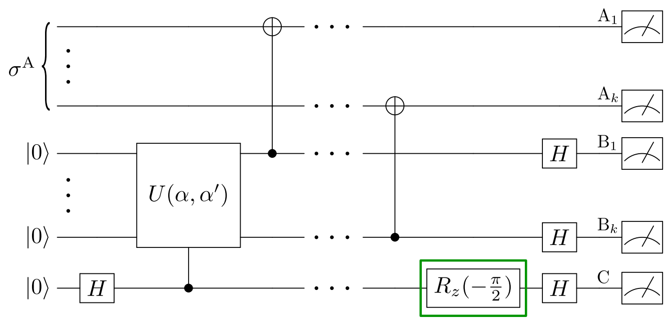

Supplementary Figure 6. Circuit to read out $\mathcal{D}(\boldsymbol{\alpha},\boldsymbol{\alpha}')$. The controlled $U(\boldsymbol{\alpha},\boldsymbol{\alpha}')$ prepares the state $|\alpha\rangle$ on the B registers when the control qubit is in the state $|0\rangle$ and $|\alpha'\rangle$ when the control qubit is in the state $|1\rangle$, so the combination of the Hadamard gate on C and the controlled $U(\boldsymbol{\alpha},\boldsymbol{\alpha}')$ prepares a superposition of the histories. The z-rotation in the green box is excluded when we calculate the real part of $\mathcal{D}(\boldsymbol{\alpha},\boldsymbol{\alpha}')$ and included when we calculate the imaginary part. The post processing is described in the text.

the Swap test [2, 3], except that we add a conditional statement.

When we exclude the z-rotation, conditioned on the control qubit C being measured in the state $|0\rangle$ we perform the Swap test between the A and B registers to get:

$$\begin{aligned} R_0 &= \mathrm{Tr}\left(\sigma^{\mathrm{A}}\left[\frac{1}{2}(|\boldsymbol{\alpha}\rangle+|\boldsymbol{\alpha}'\rangle)(\langle\boldsymbol{\alpha}|+\langle\boldsymbol{\alpha}'|)\right]\right) \\ &= \frac{1}{2}\left(\mathcal{D}(\boldsymbol{\alpha},\boldsymbol{\alpha})+\mathcal{D}(\boldsymbol{\alpha}',\boldsymbol{\alpha}')+\mathcal{D}(\boldsymbol{\alpha},\boldsymbol{\alpha}')+\mathcal{D}(\boldsymbol{\alpha}',\boldsymbol{\alpha})\right) \\ &= \frac{1}{2}\left(\mathcal{D}(\boldsymbol{\alpha},\boldsymbol{\alpha})+\mathcal{D}(\boldsymbol{\alpha}',\boldsymbol{\alpha}')\right)+\mathrm{Re}(\mathcal{D}(\boldsymbol{\alpha},\boldsymbol{\alpha}')). \qquad (8) \end{aligned}$$

If we instead condition on C being measured in the state $|1\rangle$ we perform the Swap test between the A and B registers to get:

$$\begin{aligned} R_1 &= \mathrm{Tr}\left(\sigma^{\mathrm{A}}\left[\frac{1}{2}(|\boldsymbol{\alpha}\rangle-|\boldsymbol{\alpha}'\rangle)(\langle\boldsymbol{\alpha}|-\langle\boldsymbol{\alpha}'|)\right]\right) \\ &= \frac{1}{2}\left(\mathcal{D}(\boldsymbol{\alpha},\boldsymbol{\alpha})+\mathcal{D}(\boldsymbol{\alpha}',\boldsymbol{\alpha}')\right)-\mathrm{Re}(\mathcal{D}(\boldsymbol{\alpha},\boldsymbol{\alpha}')). \qquad (9) \end{aligned}$$

Our method therefore separates the output based on the result of measuring C, and then performs the usual Swap test post processing on each partition of the output counts to get R_0 and R_1. Finally, we combine these to get:

$$\mathrm{Re}(\mathcal{D}(\boldsymbol{\alpha},\boldsymbol{\alpha}')) = \frac{1}{2}(\mathrm{R}_0-\mathrm{R}_1)\ . \qquad (10)$$

Instead including that z-rotation gives us

$$\begin{aligned} I_0 &= \mathrm{Tr}\left(\sigma^{\mathrm{A}}\left[\frac{1}{2}(|\boldsymbol{\alpha}\rangle+i|\boldsymbol{\alpha}'\rangle)(\langle\boldsymbol{\alpha}|-i\langle\boldsymbol{\alpha}'|)\right]\right) \\ &= \frac{1}{2}\left(\mathcal{D}(\boldsymbol{\alpha},\boldsymbol{\alpha})+\mathcal{D}(\boldsymbol{\alpha}',\boldsymbol{\alpha}')-i\mathcal{D}(\boldsymbol{\alpha},\boldsymbol{\alpha}')+i\mathcal{D}(\boldsymbol{\alpha}',\boldsymbol{\alpha})\right) \\ &= \frac{1}{2}\left(\mathcal{D}(\boldsymbol{\alpha},\boldsymbol{\alpha})+\mathcal{D}(\boldsymbol{\alpha}',\boldsymbol{\alpha}')\right)-\mathrm{Im}(\mathcal{D}(\boldsymbol{\alpha},\boldsymbol{\alpha}')), \qquad (11) \end{aligned}$$

conditioned on C being measured in the state $|0\rangle$. Similarly, conditioned on C being measured in the state $|1\rangle$ we find:

$$\begin{aligned} I_1 &= \mathrm{Tr}\left(\sigma^{\mathrm{A}}\left[\frac{1}{2}(|\boldsymbol{\alpha}\rangle-i|\boldsymbol{\alpha}'\rangle)(\langle\boldsymbol{\alpha}|+i\langle\boldsymbol{\alpha}'|)\right]\right) \\ &= \frac{1}{2}\left(\mathcal{D}(\boldsymbol{\alpha},\boldsymbol{\alpha})+\mathcal{D}(\boldsymbol{\alpha}',\boldsymbol{\alpha}')+i\mathcal{D}(\boldsymbol{\alpha},\boldsymbol{\alpha}')-i\mathcal{D}(\boldsymbol{\alpha}',\boldsymbol{\alpha})\right) \\ &= \frac{1}{2}\left(\mathcal{D}(\boldsymbol{\alpha},\boldsymbol{\alpha})+\mathcal{D}(\boldsymbol{\alpha}',\boldsymbol{\alpha}')\right)+\mathrm{Im}(\mathcal{D}(\boldsymbol{\alpha},\boldsymbol{\alpha}')). \qquad (12) \end{aligned}$$

Again, we combine these to get:

$$\mathrm{Im}(\mathcal{D}(\boldsymbol{\alpha},\boldsymbol{\alpha}')) = \frac{1}{2}(\mathrm{I}_1-\mathrm{I}_0) \qquad (13)$$

We also note that the controlled $U(\boldsymbol{\alpha},\boldsymbol{\alpha}')$ we have made use of here can be implemented with depth that scales linearly in the number of bits by which $|\boldsymbol{\alpha}\rangle$ and $|\boldsymbol{\alpha}'\rangle$ differ. This is accomplished by acting with X gates on all of the registers where the bit-string associated with $|\boldsymbol{\alpha}\rangle$ is 1 followed by CNOT gates from C to each of the registers where the bit-strings for $|\boldsymbol{\alpha}\rangle$ and $|\boldsymbol{\alpha}'\rangle$ differ.

Finally, we comment that reading out $\mathcal{D}(\boldsymbol{\alpha},\boldsymbol{\alpha})$ is simpler than the general case as we merely have to prepare $|\boldsymbol{\alpha}\rangle\langle\boldsymbol{\alpha}|$ (which consists of a single layer of X gates) on the B registers and perform the Swap test, without any need for or reference to C.

SUPPLEMENTARY NOTE 5 – IMPLEMENTATION CIRCUITS

Spin in a Magnetic Field

For our simulations of the spin-1/2 particle in a magnetic field, Supplementary Figure 7 shows the quantum circuit that was used on the simulator and IBM's ibmqx5 processor to perform the cost minimization and to generate the cost landscape plots (shown in Fig. 3).

Chiral Molecule

Supplementary Figure 8 shows the quantum circuit that was used on a simulator to map the cost function landscapes for the chiral molecule (shown in Fig. 4). The tunneling between the chirality states was modeled as a rotation about the z-axis by an angle θ_z. We considered the chiral molecule to be in a gas, and hence its environment is composed of other surrounding molecules that may collide with the molecule of interest. Our model for these collision interactions was implemented by performing a rotation around the x-axis by an angle θ_x (which determines the interaction strength) on an environmental qubit representing the colliding molecule, controlled by the chirality of the molecule of interest.

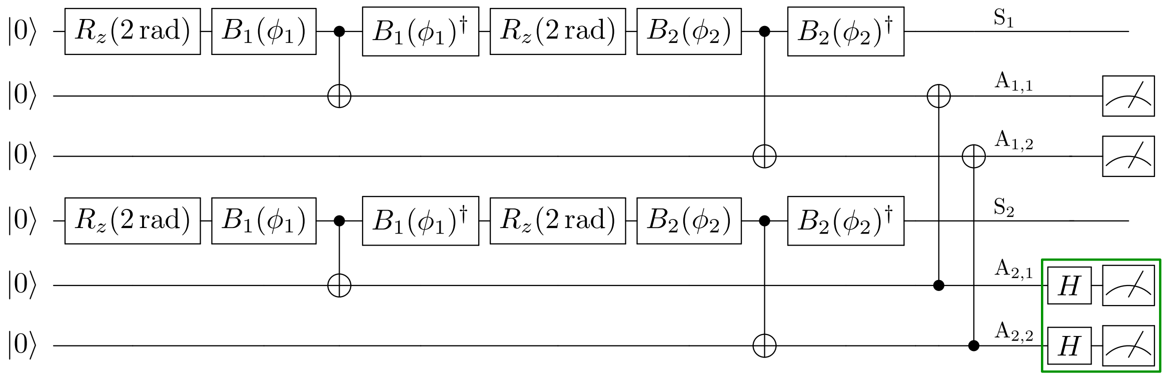

Supplementary Figure 7. Quantum circuit that we employed to evaluate the cost functions for the spin in a magnetic field. The wires labeled S represent the copies of the spin and those labeled A represent the ancillas. Note that this circuit prepares two copies of σ^{A}. The gates and measurements inside the solid green box are only included to calculate $\mathrm{Tr}((\sigma^{\mathrm{A}})^2)$, as without them this is the circuit to calculate $\mathrm{Tr}(\mathcal{Z}^{\mathrm{A}}(\sigma^{\mathrm{A}})^2)$.

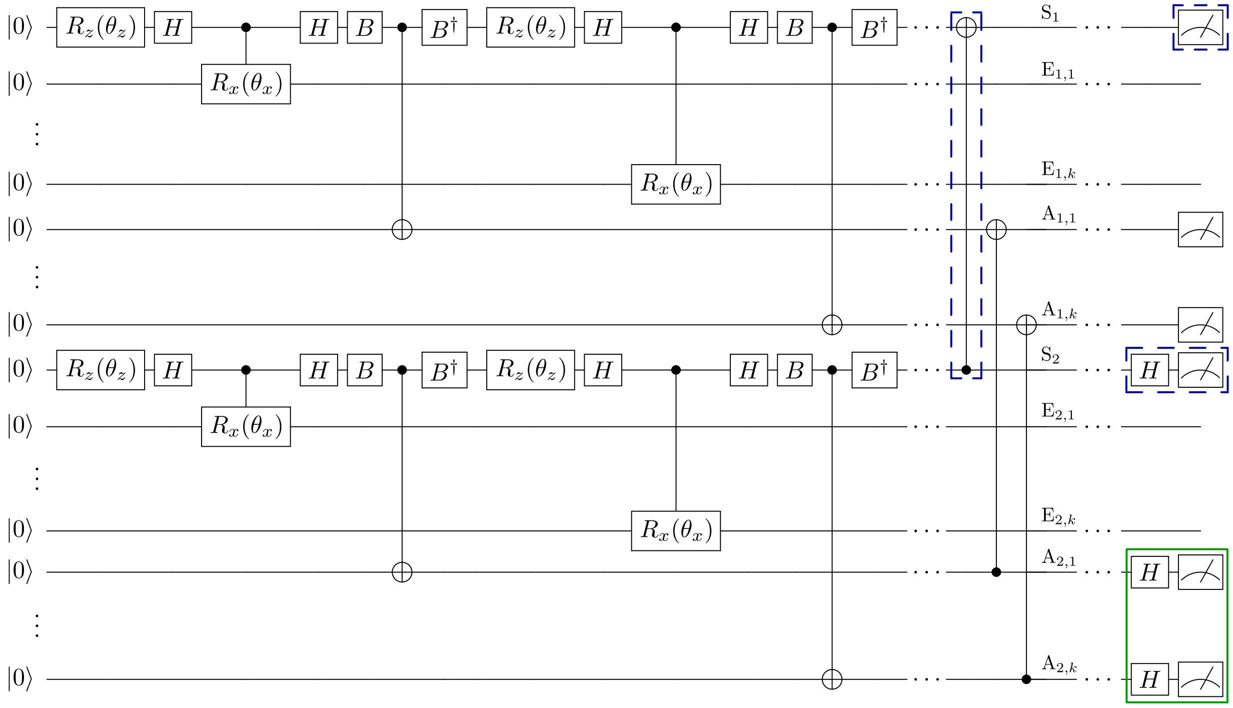

Supplementary Figure 8. Quantum circuit that we employed to evaluate the cost functions for the chiral molecule example in the main text. The wires labeled S represent the chirality degree of freedom of the molecule, E represents the environment (other surrounding molecules), and A represents the ancillas. Note that this circuit prepares two copies of σ^{SA} (and hence σ^{A}). The gates and measurements inside the blue dashed boxes are only included when we are evaluating the partial trace cost function (i.e., when working with σ^{SA} rather than σ^{A}). The gates and measurements inside the solid green box are only included when calculating $\mathrm{Tr}((\sigma^{\mathrm{A}})^2)$ or $\mathrm{Tr}((\sigma^{\mathrm{SA}})^2)$, and otherwise the circuit calculates $\mathrm{Tr}(\mathcal{Z}^{\mathrm{A}}(\sigma^{\mathrm{A}})^2)$ or $\mathrm{Tr}(\mathcal{Z}^{\mathrm{A}}(\sigma^{\mathrm{SA}})^2)$.

SUPPLEMENTARY NOTE 6 – HIGHLIGHTED APPLICATIONS

Here we provide a brief outline of two potential applications that would be viable with NISQ computers.

Spin Diffusion

The phenomenon of spin diffusion has been known for a long time [4], but an understanding of the transition from oscillatory dynamics to a classical diffusion equation as system sizes increase is still incomplete. Given that quantum dots with $\sim 30,000$ nuclear spins have been shown to exhibit spin diffusion [5], we expect this to be a very conservative upper bound on the number of spins required.

As a lower bound, simple numerical calculations that we performed show that ~ 10 spins do not appear to exhibit spin diffusion. In particular, our calculations showed that, for these small spin systems, the local magnetization does not provide a consistent family of stationary histories. (See the main text for an example of stationary histories for chiral molecules.) Note that the local magnetization forming a consistent family would be a pre-requisite for a random-walk description (and hence diffusive dynamics) of spin magnetization. Combining this lower bound with our upper bound, we expect that the transition is likely to be found with $\sim 10^2$ or $\sim 10^3$ spins.

Applying VCH to this problem would also illuminate the nature and sharpness of the transition. Namely, we anticipate that the transition will involve the disappearance of Rabi oscillations (a signature of quantum interference) for magnetization as the number of spins increases. A natural question is whether such oscillations disappear completely at a critical system size, analogous to how the chirality oscillations disappeared for the chiral molecule (discussed in the main text) at a particular decoherence rate [6]. Another possibility is that the transition is gradual, rather than sharp, and that the oscillations are merely suppressed rather than eliminated with system size.

It has been experimentally demonstrated with echo techniques that coherence is maintained during spin diffusion [7–9]. In other words, the classical diffusion equation can be understood to arise from closed-system dynamics rather than open-system dynamics, i.e., as an effect of coarse graining rather than an interaction with the environment. We would therefore only be interested in ansatzes that represent coarse grained spin information on some subset of the spins and neglect environmental effects.

Given these considerations, we can estimate the number of qubits needed to apply VCH to this situation and look for the sort of random walks that would give rise to diffusion. Let n_{total} and n_{voxel} respectively denote the total number of spins and the number of spins in the region we are following the magnetization of (the voxel). Simulating n_{total} spins requires n_{total} qubits. In order to implement the projections, we would need to have at most enough qubits to span a space large enough to account for the $n_{\text{voxel}} + 1$ possible magnetizations, though this could be coarse grained further. Therefore, to carry out this investigation for k times, we would expect to need roughly

$$2(n_{\text{total}} + k\lceil\log_2(n_{\text{voxel}} + 1)\rceil) \tag{14}$$

qubits, where the factor of two comes from the fact we need two copies of the state for VCH. Thus, our estimate for where we expect to find the transition to diffusive behavior with coarse graining translates to needing somewhere around $\sim 10^2$ or $\sim 10^3$ qubits.

Protein Folding

Proteins with up to 76 amino acids have been folded thus far using molecular dynamics simulations without adding in external forces to bias the dynamics towards the "correct" configuration [10]. However, these simulations do not include decoherence effects and are not capable of fully exploring the vast space of un-biased paths. To move beyond what can be done with these classical tools, we propose to use VCH.

In order to investigate under which circumstances a protein will follow a single deterministic path or fold by multiple paths, one could implement a quantum simulation of the process using only realistic interaction Hamiltonians and examine the histories. Conjecturing that decoherence by the environment should play an important role, we would need to consider a simulation of an initially unfolded protein as well as its environment.

Let us consider the simplified case of lattice protein folding for a chain with n_{AA} amino acids. Each connection between amino acids in such a model can be in m different configurations. This system can be represented with $\lceil(n_{\text{AA}} - 1)\log_2(m)\rceil$ qubits. In analogy with the chiral molecule example in the main text, we propose an environment model that would act with different rotations to environment qubits based on the current configuration of each connection, meaning that the size of the environment being modeled would be something like $k\lceil(n_{\text{AA}} - 1)\log_2(m)\rceil$ qubits for k times. The size of the ancillas required to record fine grained histories of this system is the same as this environmental size. Finally, given the need for two copies, we end up with a total qubit requirement of

$$2(2k + 1)\lceil(n_{\text{AA}} - 1)\log_2(m)\rceil) \tag{15}$$

qubits. For a cubic lattice with $n_{\text{AA}} = 100$ examined at 10 times, this becomes 9,660 qubits. Given that such a history analysis becomes classically intractable well before the search for the correct (native) configuration does,

we therefore think that useful instances of this application will become practical with quantum computers with between 10^3 and 10^4 qubits.

SUPPLEMENTARY REFERENCES

[1] M. Gell-Mann and J. B. Hartle. Classical equations for quantum systems. *Phys. Rev. D*, 47:3345–3382, 1993.

[2] J. C. Garcia-Escartin and P. Chamorro-Posada. Swap test and Hong-Ou-Mandel effect are equivalent. *Physical Review A*, 87(5):052330, 2013.

[3] L. Cincio, Y. Subaşı, A. T. Sornborger, and P. J. Coles. Learning the quantum algorithm for state overlap. *New Journal of Physics*, 20(11):113022, 2018.

[4] N. Bloembergen. On the interaction of nuclear spins in a crystalline lattice. *Physica*, 15(3):386 – 426, 1949.

[5] S. Adachi, R. Kaji, S. Furukawa, Y. Yokoyama, and S. Muto. Nuclear spin depolarization via slow spin diffusion in single InAlAs quantum dots observed by using erase-pump-probe technique. *Journal of Applied Physics*, 111(10):103531, 2012.

[6] P. J. Coles, V. Gheorghiu, and R. B. Griffiths. Consistent histories for tunneling molecules subject to collisional decoherence. *Phys. Rev. A*, 86:042111, 2012.

[7] Shanmin Zhang, B. H. Meier, and R. R. Ernst. Polarization echoes in nmr. *Phys. Rev. Lett.*, 69:2149–2151, Oct 1992.

[8] Torgny Karlsson, Michael Helmle, N.D. Kurur, and Malcolm H. Levitt. Rotational resonance echoes in the nuclear magnetic resonance of spinning solids. *Chemical Physics Letters*, 247(4):534 – 540, 1995.

[9] M. Tomaselli, S. Hediger, D. Suter, and R. R. Ernst. Nuclear magnetic resonance polarization and coherence echoes in static and rotating solids. *The Journal of Chemical Physics*, 105(24):10672–10681, 1996.

[10] Stefano Piana, Kresten Lindorff-Larsen, and David E. Shaw. Atomic-level description of ubiquitin folding. *Proceedings of the National Academy of Sciences*, 110(15):5915–5920, 2013.
